# Design and Validation of a Low-Cost Mobile EEG-Based Brain–Computer Interface

**DOI:** 10.3390/s23135930

**Published:** 2023-06-26

**Authors:** Alexander Craik, Juan José González-España, Ayman Alamir, David Edquilang, Sarah Wong, Lianne Sánchez Rodríguez, Jeff Feng, Gerard E. Francisco, Jose L. Contreras-Vidal

**Affiliations:** 1 Department of Electrical and Computer Engineering, University of Houston, Houston, TX 77004, USA; 2 Noninvasive Brain-Machine Interface Systems Laboratory, NSF Industry—University Cooperative Research Center for Building Reliable Advances and Innovations in Neurotechnology (IUCRC BRAIN) Center, University of Houston, Houston, TX 77004, USA; 3 Department of Biomedical Engineering, University of Houston, Houston, TX 77004, USA; 4 Department of Electrical Engineering, Jazan University, Jazan 45142, Saudi Arabia; 5 Department of Industrial Design, University of Houston, Houston, TX 77004, USA; 6 Department of Physical Medicine & Rehabilitation, University of Texas Health McGovern Medical School, Houston, TX 77030, USA; 7 The Institute for Rehabilitation and Research (TIRR) Memorial Hermann Hospital, Houston, TX 77030, USA

**Keywords:** brain–computer interfaces, electroencephalography, mobile EEG, rehabilitation, neurodiagnostics, motor intent detection

## Abstract

*Objective:* We designed and validated a wireless, low-cost, easy-to-use, mobile, dry-electrode headset for scalp electroencephalography (EEG) recordings for closed-loop brain–computer (BCI) interface and internet-of-things (IoT) applications. *Approach:* The EEG-based BCI headset was designed from commercial off-the-shelf (COTS) components using a multi-pronged approach that balanced interoperability, cost, portability, usability, form factor, reliability, and closed-loop operation. *Main Results:* The adjustable headset was designed to accommodate 90% of the population. A patent-pending self-positioning dry electrode bracket allowed for vertical self-positioning while parting the user’s hair to ensure contact of the electrode with the scalp. In the current prototype, five EEG electrodes were incorporated in the electrode bracket spanning the sensorimotor cortices bilaterally, and three skin sensors were included to measure eye movement and blinks. An inertial measurement unit (IMU) provides monitoring of head movements. The EEG amplifier operates with 24-bit resolution up to 500 Hz sampling frequency and can communicate with other devices using 802.11 b/g/n WiFi. It has high signal–to–noise ratio (SNR) and common–mode rejection ratio (CMRR) (121 dB and 110 dB, respectively) and low input noise. In closed-loop BCI mode, the system can operate at 40 Hz, including real-time adaptive noise cancellation and 512 MB of processor memory. It supports LabVIEW as a backend coding language and JavaScript (JS), Cascading Style Sheets (CSS), and HyperText Markup Language (HTML) as front-end coding languages and includes training and optimization of support vector machine (SVM) neural classifiers. Extensive bench testing supports the technical specifications and human-subject pilot testing of a closed-loop BCI application to support upper-limb rehabilitation and provides proof-of-concept validation for the device’s use at both the clinic and at home. *Significance:* The usability, interoperability, portability, reliability, and programmability of the proposed wireless closed-loop BCI system provides a low-cost solution for BCI and neurorehabilitation research and IoT applications.

## 1. Introduction

Since the early 1960s, when electroencephalography (EEG) data were first digitized and processed with a computer to today, much progress has been made in harnessing the potential of brain–computer interface (BCI) applications [1,2]. While EEG measurements are affected by many factors, including physiological and non-physiological artifacts [3] resulting in low signal-to-noise ratios [4], recent advancements in de-noising (e.g., [5,6]) and deep learning [7,8] techniques have driven the emergence of viable clinical and non-clinical BCI applications based on scalp EEG [1,2,9]. These applications include, but are not limited to, seizure state prediction [10,11], sleep stage analysis [12], cognitive workload assessment [13], motor-imagery-based brain–computer interface (BCI) systems [14,15], neurorehabilitation [16,17], multi-modal and multi-brain–computer interfaces [18], brain-controlled vehicles [19], EEG-based home control [20], virtual reality [21], and interactive virtual environments [22]. While the future of these proof-of-concept BCI-enabled applications is promising, there are a number of technical challenges that remain before the widespread translation and adoption of these systems is realized.

**Figure 1:**
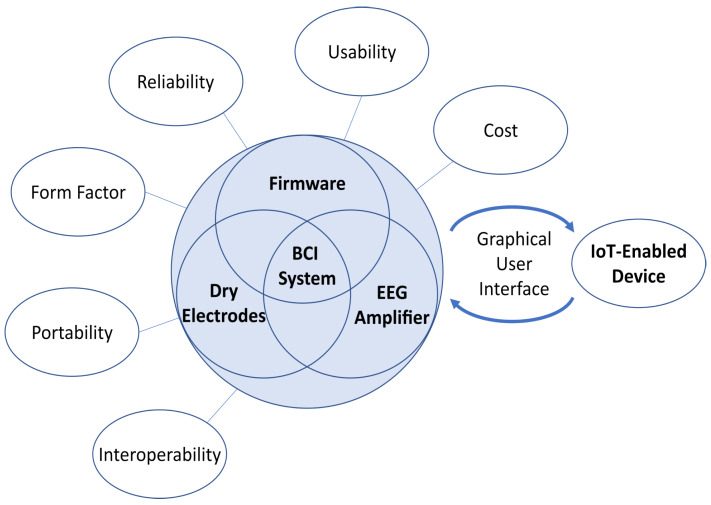
Design criteria adopted in this research to maximize the translational impact of noninvasive (non-surgical) closed-loop BCI technology (adapted with permission from [23]).

Prior efforts from the scientific, engineering, medical, regulatory, industrial, and patient-advocate communities [23,24,25,26] have addressed the challenges and opportunities for accelerating the translation of closed-loop BCI systems for medical applications. Some of the key challenges identified in deploying these technologies to end-users include usability, interoperability, accessibility, and mobility, as well as the lack of standards (device, performance, clinical, and end-user metrics). For example, current commercial EEG amplifiers and BCI headsets are prohibitively expensive, lack interoperability, or fail to provide a high signal quality or closed-loop operation, which are vital for BCI applications [23]. To address these challenges and facilitate the translation of BCI systems, we adopted criteria derived from the above stakeholder meetings for the design of closed-loop BCI systems (Figure 1). Next, we briefly review these criteria. The reader is referred to the source publications from these stakeholder meetings for additional details.

Portability [27] and interoperability both affect the type of BCI applications that can be considered. Most commercial EEG systems are tethered to immobile processing hardware, making them difficult to deploy outside of the clinic or laboratory. A portable and wireless EEG system is highly preferred so it can be used outside lab and clinical settings in clinical and non-clinical mobile applications at home, work, or play. Additionally, a system design that only provides control of a single device or the analysis of a single protocol significantly limits the potential for BCI systems, so a generalized control or analysis framework is preferred over a device-, task- or protocol-specific system to maximize interoperability in the widest sense.

Usability [28,29], form factor [30], and reliability [31] all significantly affect the user’s experience. The current commercial EEG systems are generally difficult to set up and use, particularly in medical applications by users with disabilities. This is a critical challenge for applications that will be used by the public as a complex system setup may be too difficult or take too long for an untrained user to operate without technical or expert assistance. A difficult challenge in the design of an EEG headset is accommodating the many different head sizes and shapes, hair types and styling, and user preferences, but designing many different variations may not be economically feasible nor desirable for a commercial system. While a one-size-fits-all design is preferable, the ability for the system to be adaptable must be emphasized early in the design process and heavily tested in ecological settings. Moving this technology to low-cost hardware will increase accessibility, but, if the system is not reliable, the resulting user frustration may lead to product abandonment. Therefore, extensive software and hardware bench testing must be performed to ensure reliability.

Outside of factors that affect the design considerations and the user’s experience, the ability for the system to process EEGs quickly and effectively is a necessary condition for complex closed-loop BCI applications. This necessity is due to the fact that EEG suffers from a low signal-to-noise ratio, low spatial resolution, and high prevalence of artifacts, such as eye movements, eye blinks, and motion artifacts [32], to name a few. Many of the commonly used signal de-noising methods are not suitable for real-time or mobile applications [5,6], so the selection of on-chip real-time signal-de-noising methods is a crucial decision that should be considered early on in the development process. Once the EEG signals are de-noised, a neural decoder or neural classifier is commonly employed to extract valuable information, e.g., motor intent, emotional state, or other classes of internal states, from the brain signals acquired with EEG [7,33]. However, most current EEG systems do not provide the decoding functionality necessary for implementing closed-loop BCI applications without additional hardware and software. The above challenges provided the motivation for the development of the proposed EEG-based closed-loop BCI headset.

While there are low-cost commercial dry EEG amplifier systems available on the market, none meet the criteria outlined above in Figure 1. For example, the Ultracortex Mark IV EEG headset from OpenBCI [34] is a popular open-source EEG headset design and is sold for a relatively low cost ($399.99 for the user to 3D print the headset, $899.99 for the 3D-printed and assembled version at the time of publication). However, each headset electrode holder must be manually manipulated for each user, which is not as user-friendly as a design that employs a single manipulator for headset adjustments. Additionally, the OpenBCI headset does not provide processing onboard with the amplifier. Instead, it requires a separate computational unit for signal processing. The Muse 2 system [35] is one of the lowest-cost commercial amplifiers available ($249.99) and includes a software application that provides standard biofeedback. A major drawback with the Muse 2 system is that an annual subscription must be purchased to use many of the available software features. Additionally, the Muse 2 system only has two forehead sensors and two sensors located behind each ear, which limits the potential applications for systems based on this system. Like the OpenBCI Mark IV headset, the Muse 2 system does not have onboard processing capabilities, meaning a separate computing unit must be employed. In another example BCI system [36], the researchers designed specialized dry EEG electrodes for a low-channel-count EEG system for steady-state visual evoked potential (SSVEP) applications. The main focus was to validate the dry-electrode design, so the authors used a relatively expensive commercial amplifier (NeuroScan Synamps, CompuMedics Neuroscan, Victoria, Australia). In another study [37], a low-cost system integrating EEG and augmented reality (AR) capabilities was deployed for SSVEP-based applications. Instead of creating a custom amplifier, the authors opted for a low-cost two-channel EEG system for signal acquisition (EEG-SMT, Olimex, Plovdiv, Bulgaria). In [38], the authors developed an inexpensive BCI system for upper-limb stroke rehabilitation. This system relied on a higher-cost Emotiv (Emotiv Epoc+, Emotiv, San Francisco, CA, USA [39]) commercial amplifier and utilized open-source functionality from BCI2000 [40], without a dedicated user-friendly interface. While the market for commercial EEG amplifiers is expanding, there are no suitable commercial systems that meet the specifications required for more closed-loop BCI applications. For a recent review of portable EEG devices with wireless capability, see [41].

The rest of the paper is organized as follows: Section 2 will describe the methods, including hardware and software selection and development, as well as the methodology for system validation using bench testing and human-subject experiments in the laboratory, clinic, and home. Section 3 presents the results of the system validation tests, including first-in-human validation in an ecological setting. Section 4 provides a discussion on crucial design decisions and the development of the system generally. We conclude with some lessons learned and next steps.

## 2. Methods

The design criteria were based on the recommendations from stakeholder meetings [23,24,25,26,42]. The design choices based on the design factors shown in Figure 1 will be discussed in detail through the following sections.

To define the product and the engineering specifications for the system, we parcelled these target specifications into four key areas: the headset specifications for a universally fitting design, the desired characteristics for the EEG amplifier and sensors for artifact detection, and the specifications for the brain–computer interface itself. These specific engineering requirements are detailed in Table 1. The following section will detail the user-centered design of the headset, the development of the software, and the approach followed for bench testing and experimental validation with human participants for the system.

### 2.1. Headset Design

Proper headset fit for the users is a critical factor affecting the system’s performance, usability, and comfort, but most headsets on the market do not fit as well as research-grade soft EEG caps [43,44]. Traditional soft EEG caps are still the most widely available option in terms of accommodating both head size and shape variations [45,46]; however, they have some disadvantages compared to a headset: (1) Disinfection: Headsets can be disinfected by surface cleaning while EEG caps need to be immersed, after removing the electrodes, into a disinfection solution for several minutes; (2) Donning/doffing: Headsets are usually faster to set up than EEG caps, which may require assistance, particularly if based on wet electrodes; (3) Electrode localization: Headsets can help to maintain correct electrode positioning while EEG caps may result in electrode displacements from session to session; (4) Fitting: Headsets typically have a mechanism for fitting head shape and size, whereas EEG caps need to be selected in some discrete ranges varying from small to extra-large, which may lead to poor electrode set-up in some cases as head size variations are continuous; (5) Form factor: Headsets may be more desirable in terms of the aesthetics than EEG caps; (6) Single-hand use: Headsets may allow single-hand use for donning/doffing, which may be critical for users with hemiparesis or other hand disabilities. Overall, the wide range of variations in human body biometrics demands flexibility and adjustability in designing a more accommodating headset. Anthropometry data are widely used as a reference of variations to design products with optimized fit, comfort, functionality, and safety [47]. In terms of size management, there are two different approaches. One approach is to offer the headset in different sizes to fit a wide range of users. Another approach is to offer a single size with adequate adjustments in multiple degrees to fit all users. Previous research in the development of a one-size-fits-all headset has found success, providing support for this approach [48,49].

One important requirement in the design of mobile devices is the need for single-handed device interaction as the headset will likely be used by people with a limited attention span and upper-limb and/or hand impairments, including reduced mobility and hand dexterity (e.g., older individuals and persons with chronic stroke [17]). These physical limitations significantly influence the details of the design, the mechanical controls, and the overall form factor. As indicated in other studies [50], the hardware design influences the user’s interaction with the device. For this reason, the design process should include a detailed ergonomic evaluation to ensure all controls are intuitive for one-hand use.

As a device to be used directly by consumers, general usability factors should be considered and optimized, including the overall weight, adjustability, operational clarity and accuracy, user comfort, and aesthetics [51]. Additionally, a good fixation of the scalp and skin electrodes should be provided for reducing the contact impedance at the electrode–scalp/skin interface, which enhances the signal-to-noise ratio [46].

#### 2.1.1. Electrodes

The headset design process began by selecting the locations of five EEG channels. Five electrode locations (Frontocentral locations: FC3, FC1, FCz, FC2, and FC4) were selected with a reference to the international 10–20 system provided by the American Clinical Neurophysiology Society guidelines [52]. These were selected based on the proximity to the primary motor cortex and the effectiveness of using these electrodes for motor-related BCI paradigms, including motor imagery classification [53] and movement-related cortical potential (MRCP) identification [17]. The authors note that the electrode locations can be modified within the 3D headset model for paradigms that require EEG collection from other areas of the scalp.

Dry EEG comb electrodes with 5 mm extended prongs (Florida Research Instruments, Inc., Cocoa Beach, FL, USA) were selected for this device to maximize the usability and shorten the set-up time. Comb electrodes [54] are recognized as an effective solution for collecting EEGs through longer-hair conditions and the selected electrodes end in blunt tips for long-term wearing comfort. While these dry electrodes alone will likely go through users’ different hairstyles and/or hair types to reach the scalp, without a specific mechanism to secure and maintain a constant steady contact during use, it is still likely to fail the needs of most users and needed to be addressed during the design of the EEG electrode holders.

An additional functionality of the headset is the capability to measure eye movements and eye blinks using electrooculography (EOG) sensors, whose outputs could be used for real-time de-noising of the EEG signals or even as additional control signals. Ancillary experiments (to be reported elsewhere) provided support that three EOG sensors can be used to effectively extract information about eye blinks and eye movements in the vertical, horizontal, and oblique axes. These EOG sensor locations are located at the right temple, the left temple, and directly above the participant’s left eye.

Two electrodes, one behind each ear, complete the set of electrodes/sensors available in the headset. The skin sensors are adjustable in position and orientation to adapt to and fit a wide range of face profiles and contours while maintaining a constant and steady contact.

#### 2.1.2. EEG Electrode-Holder Design

**Figure 2:**
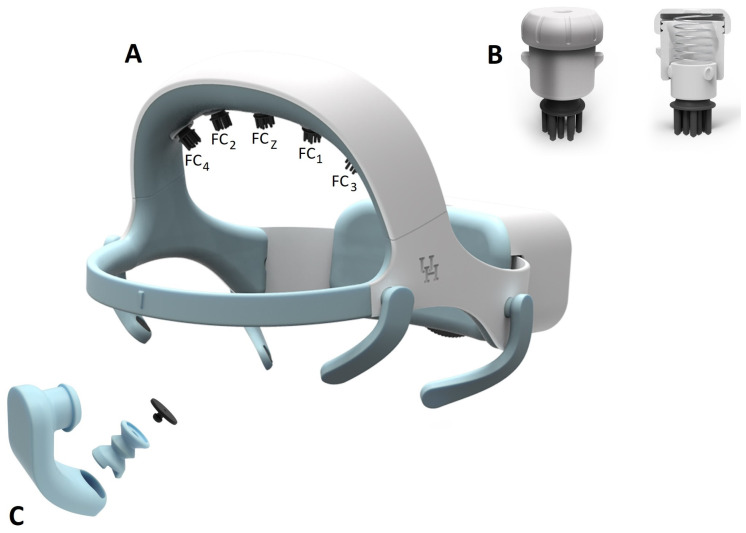
(**A**) Fully assembled one-size-fit-all (patent pending) headset design. (**B**) Dry-electrode bracket. (**C**) The skin sensor holder. This figure was adapted from US provisional patent #62857263.

One challenge for mobile EEG systems is to secure the electrodes and obtain good impedance for recordings. This is particularly important when using dry electrodes that cannot benefit from the viscous gel typically employed in wet-electrode systems. For the dry electrodes that are placed over the user’s hair, it is common to experience unstable and noisy signals due to poor or intermittent contact between the electrode and the head scalp [55,56]. To meet this challenge, a unique self-positioning dry-hair electrode holder was developed, as shown in Figure 2B. The holder is a proprietary (patent-pending) design for holding the designated electrode while providing a self-positioning rotational mechanical linkage that helps facilitate hair penetration of the electrode tips. The holder is 1.7 cm in diameter and 1.9 cm in height and is composed of three parts: the slider, the housing, and the cap. A screw-and-nut pair is used to fasten the electrode tip to the lower end of the slider. The fully shielded electrical wire is oriented between the screw and the electrode’s inner wall. The wire is routed through the center open space and the center hole on the cap. The slider is spring-loaded with a vertical travel of up to 10 mm. The electrode will move up and down along three spiral tracks, which allows for rotation of up to 120 degrees, to accommodate the regionally changing head shape. This rotation will assist the electrode tip in moving through the user’s hair for improved contact with the scalp. The spring will help to maintain a constant pressure between the electrode and the contact surface. The headset and electrode tip design are covered by US provisional patent #62857263.

#### 2.1.3. EOG Electrode-Holder Design

The headset system includes three electrooculography (EOG) sensors to track the users’ eye movements. Two sensors are positioned at the temple area along the side of each eye and a third is positioned directly above the user’s left eye. Typically, EOG skin sensors require the application of a conductive gel medium or tape to achieve steady constant contact with the skin. This headset is designed with accessibility for individuals with limited dexterity, so it is undesirable to use sensors that require gel or tape. For that reason, the headset uses dry skin sensors.

A proprietary EOG sensor-holder arm was developed to maintain a constant contact with the skin. The holder is composed of two parts: an arm and an EOG sensor plug (Figure 2C). The EOG sensor sits in the socket of the plug and is wired through the hollow arm, which is connected to the main board. The arm is printed in a medical-grade skin-safe flexible resin and is designed with a unique structure and form that makes it flexible while maintaining a constant pressure at the tip. The EOG plug is formed similarly to accordion pleats, which makes the plug compressible and can be flexed in any direction. The plug sits in an opening at the tip of the arm with an interference fit. The arm is rotatable around the connection on the structure to handle variations in face contours between users. The sensor plug’s spring motion applies a constant pressure to the skin surface to maintain a steady contact.

#### 2.1.4. Headset Size and Adjustment Mechanism Design

Anthropometric data [57] were used to determine the overall device size in relation to the range of head size variations. The sizing parameters are referenced from the measurements of the smallest (5th percentile female) to the largest (95th percentile male) head sizes. The key dimensions in design consideration are the head breadth, circumference, and length. The size range in three dimensions provides a guide for the design of the adjustment mechanisms. The differences in head breadth, circumference, and length between the 5th percentile female and the 95th percentile male are 2.7 cm, 8.9 cm, and 4.1 cm, respectively. A digital mannequin corresponding to the 5th percentile female was developed and then scaled up to the 95th percentile male. These two digital mannequin models served as the basis to build the headset model in a 3D digital SolidWorks environment.

Traditional anthropometry calculation is based on a uniform variation in several dimensions. For instance, if the head length increases, the head breadth is expected to also increase by a consistent ratio. In some cases, the head breadth and the head length do not follow the common ratio due to unique head forms. This characteristic was confirmed with the real-world data collection for this study, which helped to determine a more realistic range of deviation. Due to this discrepancy, the head-breadth-adjustment mechanism was designed to be independent of the head-length-adjustment mechanism. Based on the electrode mapping and the general mechanical adjustment concept, an initial headset structure was developed, which includes 3-degree of freedom adjustments with a sufficient range to fit 90% of all users.

The final design (Figure 2A) utilizes a large dial (6.5 cm in diameter and 0.4 cm in thickness) in the back to adjust the overall circumference. The end of the ear-hub band is designed with gear teeth in a slot along the center line. The left and right band overlap in the electrical box where they connect to the dial through the gear. The outer perimeter of the dial is shaped with fine convex diamond textures. The dial protrudes 0.6 cm out of the box and is designed to be turned easily in both directions with one finger. The dial’s clockwise rotation will extend two ear-hub parts to increase the headset circumference, whereas the counter-clockwise rotation will contract two parts to reduce the circumference. The overall circumference adjustment range is up to 8.9 cm. With a unique semi-flexible structure design, the headset is a one-size-fits-all solution.

#### 2.1.5. Headset Fabrication

The 3D model for the headset was designed with SolidWorks (SolidWorks 2019, Dassault Systemes, Vélizy-Villacoublay, France) and prototyped with a 3D printing process. Two types of printers were used in producing the prototype. An Artillery Sidewinder X1 FDM printer (manufactured by Shenzhen Yuntu Chuangzhi Technology Co., Ltd., Shenzhen, China) was used for the rigid-structure printing, while a Saturn resin printer (manufactured by ELEGOO technology Co., Ltd., Shenzhen, China) was used to print the flexible components. Two medical-grade thermoplastic resins were selected for the primary headset components: Taulman Nylon 910 (produced by Taulman3D Material, Linton, IN, USA) and Flexible 80A resin (produced by Formlabs in Somerville, MA, USA). The Taulman Nylon 910 resin was used to build the rigid structural parts of the headset as it has similar strength and stiffness to polypropulene (PP) and is FDA-approved for skin contact, and the parts can be repeatedly bent while still returning to the original shape. The Flexible 80A resin was used to build all elastic parts and is also FDA-approved for skin contact. The resulting flexible headset parts are stiff but soft with an 80A shore durometer. In addition to these two primary resins, two additional resins were used for the internal components. Esun PLA+ was used to fabricate the rear adjustment plate and dry EEG brackets while Polymax PC resin (Polymaker, Shanghai, China) was used to fabricate the ratchet gear and adjustment dial. The finalized design is presented in Figure 2. From an aesthetic standpoint, an emphasis was placed on creating a headset with clean and smooth external surfaces.

### 2.2. Design of the BCI Module

The following subsections detail the hardware and software component selections and development for the BCI module.

#### 2.2.1. Hardware Selections and Development

The primary hardware considerations of the BCI module include the selection of the processing unit, the design and manufacturing of the custom amplifier, and the power system.

##### Processor Selection

The BeagleBone Black—Wireless (BBB-W) [58] was selected as the BCI processor for its low cost, availability, compatibility, and WiFi capabilities. Moreover, the availability of an open-source LabVIEW toolkit (LINX LabVIEW [59]) significantly reduced the software redesign. The BBB-W has a 1 GHz ARM processor, 512 MB of DDR3 RAM, and 4 GB of onboard storage, providing the computational power and storage space necessary for the BCI headset.

##### Design of the Integrated Amplifier and Processing Board

**Figure 3:**
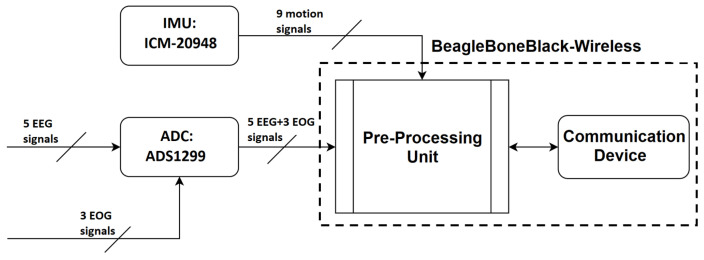
Block diagram of the EEG amplifier board.

In EEG systems, an instrumentation amplifier acts to increase the amplitude of the detected signal to a level that can be further processed while an input buffer amplifier eliminates the need for impedance matching. Recently, the term amplifier has been broadened to also include the digitization of the analog signal through an analog-to-digital conversion (ADC) chip, wireless communication, and motion-detection system. In the proposed BCI system, there are three main components on the amplifier board: signal amplification, analog-to-digital conversion, and motion sensing. Following the ADC step, it is necessary to pre-process the signals before transmission to the processing unit. These steps are summarized in Figure 3.

With respect to the amplifier, there are some electrical characteristics that are expected with any EEG amplifier [60]. The ADS1299 chip from Texas Instruments (Dallas, TX, USA) [61] was selected as it best matched the intended functionality. Its characteristics are summarized in Table 1—section Amplifier Characteristics. The minimum requirements for the inertial measurement unit (IMU), which provides motion sensing, were low energy consumption, a digital signal with more than 10 bits resolution, and the inclusion of a 3-axis accelerometer and a 3-axis gyroscope. Table A1 in the Appendix A section presents the characteristics of the ICM-20948 [62], which was selected because of its low error, its low power consumption, and the availability of a magnetometer.

For communication between the amplifier and the processing board, either an integrated approach or a system that relies on Bluetooth for communication between these modules must be selected. Rather than develop independent amplifier and processing board hardware modules that would communicate over Bluetooth, the possibility of missing data packets in this crucial stage, Bluetooth’s line-of-sight requirement, and the computational capabilities of the BBB made an integrated amplifier and processing unit more desirable. For this combined unit, the serial peripheral interface (SPI) communication protocol was employed for communication between the processing unit and the directly connected amplifier.

##### Power System

The BBB amplifier is powered by a relatively small 3.7 V battery (BatterySpace p/n: PL-383562-2C single cell Polymer Li-Ion 3.7 V/800 mAh/2.96 Wh, 64 mm × 36 mm × 4 mm/ 18 g, UL listed, UN approved battery) because portability was an important design factor [63]. Based on the maximum expected power consumption of 1.48 kWh from our system due to signal processing and constant communication with an external device (e.g., smart phone or tablet), the battery guarantees at least two hours of use. For charging of the battery, the procedure described in the “Battery Power Source/Charger” section of the OSD3358 Application Guide [64] was implemented for the system.

#### 2.2.2. Software

For the development of the device, LabVIEW (National Instruments Inc., Austin, TX, USA) was selected as the primary coding language due to its extensive libraries and access to National Instruments’ hardware and software in the early phases of the design. We note, however, that any coding language could instead be used with the selected hardware, and, in fact, a C++ version of the BCI firmware module has also been developed. This section details the main considerations, modular design, and resulting open- and closed-loop characteristics for the system software. The primary focus throughout the software development was on maintaining real-time capability, modularity, and flexibility to implement different BCI applications, thereby increasing the interoperability of the system.

##### Firmware

While LabVIEW real-time toolkits can sample at a constant frequency, this functionality requires the National Instruments onboard hardware clock, so setting a constant sampling frequency through LabVIEW is not possible on third-party processing boards. The firmware designed for the system instead employs spline interpolation, so the system can sample EEG and EOG at a rate set by the user, limited only by the computational power of the processing board. We have also developed a faster C++ implementation that does not require interpolation.

##### Communication

The BeagleBone Black—Wireless (BeagleBoard.org Foundation, Oakland Charter Township, MI, USA) processing board has both WiFi and Bluetooth capabilities (802.11 b/g/n WiFi and Bluetooth 4.1 plus BLE), which are important for the goal of creating a completely portable system. This gives the BCI device the capability of communicating with any device that can be controlled remotely. In addition to communicating with WiFi-enabled devices, to remain completely portable, the device includes a user interface that communicates with the system through the available LabVIEW web service. For the design of this interface, HyperText Markup Language (HTML), Cascading Style Sheets (CSS), and Javascript (JS) were selected as the base languages for the interface, since they can be used for the creation of a cross-platform interface that can be accessed from any browser and display that can handle the computational demands of the system.

##### Open-Loop Capabilities

The BCI device can be used to collect and save raw data from a user according to an easily modifiable protocol. These data include five EEG channels, three EOG channels, and accelerometer data from the IMU. Due to the design considerations, the maximum sampling rate that can be achieved for raw data collection and saving is 80 Hz. To achieve this sampling rate, the system utilizes LabVIEW’s point-by-point virtual instruments and channel mechanisms. Sampling up to 80 Hz means future applications can be developed that require a spectral analysis of the Delta, Theta, Alpha, Beta, and lower Gamma frequency bands.

##### EEG De-Noising Capabilities

We implemented various real-time de-noising capabilities, including spline interpolation, low-pass filters, high-pass filters, and an H-Infinity adaptive noise cancellation filtering framework. Spline interpolation provides a mechanism to handle any lost data packets as well as the ability to maintain a constant sampling frequency, a requirement for accurate filtering. The low- and high-pass filters allow for the isolation of frequency bands, a method that can be used for the spectral analysis commonly found in EEG signal-processing paradigms. The H-infinity filter employs data collected from the three EOG sensors in the automatic real-time removal of eye movement and eye blink artifacts [5], which is one of the most common biological artifacts affecting EEG. In addition, it can detect and remove amplitude drifts and recording biases simultaneously [5]. A recent extension can identify and remove motion artifacts as well [6].

##### Closed-Loop Capabilities

To test the closed-loop capabilities of the system, an example experimental protocol was implemented. This experimental protocol includes a real-time signal processing pipeline, training data collection, training of a machine learning model, testing of the trained model in real time, a graphical user interface (GUI), and constant communication with a third-party device. Due to design considerations, the system processes EEG and EOG data at 40 Hz and can save data at 20 Hz while simultaneously processing the signal, controlling a third-party WiFi device/object, and controlling a user interface over the web server. Sampling at up to 40 Hz supports applications that require a spectral analysis of the Delta, Theta, Alpha, and lower Beta bands. The authors note that further coding optimization effort could be made on the firmware design, which would likely allow for higher sampling frequencies.

##### Modular Software Design

While specific experimental protocols can influence the overall system software design, there are several key modules that will appear in many BCI systems. These common modules include an impedance check to assess the signal quality, a module for implementing the data-collection parameters and machine learning model training, a module to allow for user feedback through a survey mechanism, and a module for user help and troubleshooting. Additionally, as the system is designed to be used both inside and outside of a clinical setting, an extensive debugging user interface is necessary. The authors emphasize that the current system software is designed to be modifiable for many BCI applications.

*Aesthetic Design of the User Interface—*There were several aesthetic choices made during the user interface development that helped to further enhance the usability of the system. Colors and sizes were optimized to account for possible vision deficits by end-users. This includes large font sizes and components for those with poor vision and a color-blind-friendly design [65]. The development focused on hemianopia- and nystagmus-friendly design features, such as the button and icon designs, the logo position as a reference point, easing the cognitive workload, and creating a simple but appealing design [66].

*Impedance Check—*Ensuring signal quality involves measuring and displaying impedance values for the user so that, for electrodes that show high impedance values, the user can adjust the electrodes accordingly. Real-time display of these impedance values is therefore an essential module for BCI systems. Here, the module is designed to set up the amplifier, interpolate at a constant sampling frequency, filter at the prescribed subband range (as designated by the ADS1299 documentation), and send the resulting impedance values to the user interface in real time.

*Model Calibration—*For applications that rely on machine learning model predictions for the acquisition of a control signal, training data must first be collected to train the machine learning model. The system allows for customization of the protocol for different BCI paradigms. Functionality has been built to allow for the acquisition of multiple days of data, which can then be used to train a machine learning model or monitor task performance and progress. As an initial machine learning model selection, the system includes a support vector machine (SVM) library (including hyperparameter optimization and n-fold cross validation), which the user can initiate from the user interface. Once the SVM model is trained, the user is then able to proceed with the model-testing stage. In addition to collecting EEG data for each testing trial, this module also collects protocol-specific characteristics, which can be analyzed later by a clinician or researcher to verify the progress of a user through a specific protocol. The authors note that, while only an SVM library has been developed, many types of machine learning model can be implemented in the device within the limits of the available onboard memory.

*Survey Collection—*The proposed system includes a survey functionality that gives the user a way to provide feedback, which can be completed at any time. These results are stored onboard the processing unit for further analysis. This pop-up interface is presented in Section 3.3.1 , which can be modified depending on the type of feedback desired for a particular application.

*Debugging Interface—*For ease of use, significant effort was made in developing a debugging user interface. The device’s debugging interface, presented in Section 3.3.1, includes mechanisms to check whether the internal LabVIEW script is running, whether the web server is correctly activated, a signal-impedance check with a channel-selection mechanism, and a device-communication check. This provides the user with a series of simple steps that can be performed without guidance to address potential system faults. The debugging home screen provides the user with easy-to-understand descriptions of each debugging page to make troubleshooting as painless as possible.

### 2.3. System Validation

To demonstrate the features and functionality of the system, assessments were designed to validate three key areas: the headset design, the open-loop capability of the system, and the closed-loop capability of the system (see Table 2).

All tests were performed either at the University of Houston (UH) under a human-subjects protocol approved by the Institutional Review Board (IRB) at UH (IRB studies #3430 and #2515) or at the University of Texas Health Science Center of Houston (under IRB study HSC-MS-20-1287). Five neurologically intact adults (four males and one female) were recruited and underwent a series of tests for validation of the headset design and open-loop BCI functionality. One 66-year-old male participant with chronic stroke, with hemiparisis on the left side of his body, participated in the validation of the closed-loop functionality during at-home use. All recruited participants gave their written informed consent prior to testing.

#### 2.3.1. Headset Design Validation

Usability testing was conducted to validate the headset design. The testing focused on two key aspects: the overall participant comfort of the system during extended periods of use and the overall usability of the system based on the System Usability Scale (SUS) [28,68]. These tests were carried out with a diverse set of participants with varied head sizes, shapes, and hair types.

#### 2.3.2. Open-Loop Brain–Computer Interface Validation

To evaluate the functionality of the BCI, a set of tests was performed that focused on the performance of the BCI in open-loop operations, impedance measurement, EOG measurement, and the synchronization of EOG, EEG, and head-movement data in real time.

#### 2.3.3. Closed-Loop Brain–Computer Interface Validation

**Figure 4:**
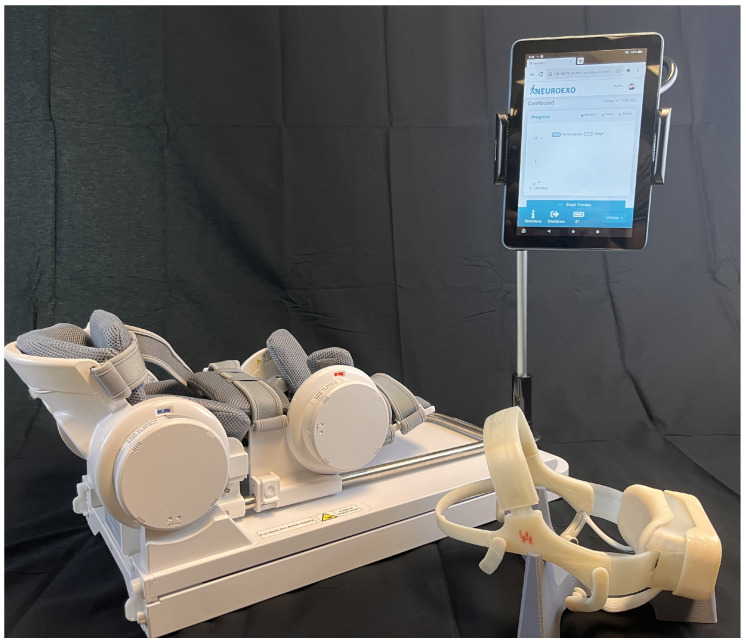
A custom EEG-based BCI headset with wireless tablet-based (Fire 8, Amazon, Seattle, WA, USA) graphical user interface (GUI) and an IoT-enabled powered upper-limb exoskeleton robotic device (Rebless, H Robotics, Austin, TX, USA) deployed in a sample neurorehabilitation application.

To assess the closed-loop capabilities of the system, an example deployment application from the neurorehabilitation literature was selected [17]. Specifically, a BCI–robot system, including an IoT-enabled robotic device and a tablet with a custom graphical user interface (GUI), is presented as an example of deployment in a neurorehabilitation application; see Figure 4. This specific implementation was chosen based on previous research on a closed-loop BCI for rehabilitation [17]. In their study, the authors developed a BCI system for upper-limb rehabilitation after stroke that focused on detecting motor intent to control a motorized exoskeleton for the upper limb. They achieved this by identifying a movement-related cortical potential (MRCP) that precedes voluntary movements of the upper limb (e.g., readiness potential). This type of cortical potential has been extensively studied [69,70,71,72,73,74] as a means of predicting motor intent. However, other brain features, such as changes in EEG rhythms, could be used to detect motor intent.

In that study [17], the authors utilized an expensive high-density EEG system and a custom motorized upper-limb exoskeleton supervised by a team of trained technicians and physical therapists to conduct the clinical trial in stroke survivors. Encouraging clinical results were observed, with all participants showing sustained improvements in motor abilities following the cessation of the rehabilitation protocol. These positive outcomes, along with the necessity for increasing accessibility, usability, interoperability, and mobile deployment at home made this example application suitable for validation of the proposed BCI headset. In addition to the development of the system itself, this example deployment required the collection of data both in the clinic and at the participant’s home, which allowed for an assessment of the system’s usability outside of the clinic.

## 3. Results

### 3.1. Headset Design Validation Results

In this section, we report the results from the system comfort and system usability scale assessments. These assessments were carried out to validate the final designs for the overall headset and the electrode holders.

#### 3.1.1. System Comfort Test

Table 3 shows the system comfort results from five participants with a diverse range of head shapes, sizes, and hair types. Participants responded to the following questions: “Did the headset move during the study?”, “Did the headset cause the sensation of dents on your head?”, “Did the headset feel too big on your head?”, and “Did the headset feel too small on your head?”. The participants could choose from the following rating values: “Strongly Agree”, “Agree”, “Neutral”, “Disagree”, and “Strongly Disagree”. Although the overall level of comfort across the participants was high (e.g., 4.6/5 for three of the questions), two reported reduced comfort in one item due to the occurrence of feeling of dents on their scalp after two hours of use.

During this assessment, it was confirmed that when a female participant whose head measurements matched the fifth percentile of female head sizes wore the headset, the headset was in its fully contracted state with a comfortable and secure fit. When repeating this assessment with a participant near the 95th percentile of male head circumference, the headband’s vertical sizing mechanism expanded 1.9 cm on both sides to accommodate the larger distance between the top of the head and the ears.

#### 3.1.2. System Usability Test

The SUS [28] was used to assess the usability of the system. This metric has been employed previously in the assessment of usability for other BCI systems [67,75]. For the proposed system, the average SUS score among the five participants was 90.5, which is above the threshold (65) for an acceptable system [67]. All participants were able to independently and intuitively don the headset with only one hand.

### 3.2. Open-Loop BCI Validation

In this section, we report the results from the signal quality, EOG collection, IMU synchronization, and open-loop BCI assessments.

#### 3.2.1. Signal-Quality Test

**Figure 5:**
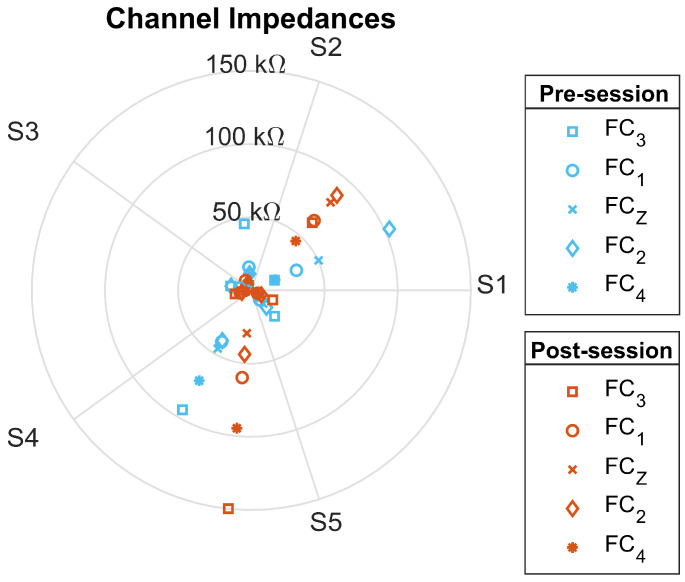
Channel Impedance: Impedance values from the open-loop sessions for five participants. The values were taken before (blue) and after (orange) the session. The values are in kΩ.

The impedance values from all electrodes were collected before and after the open-loop BCI test. The beginning and final impedance values for each electrode are presented in Figure 5. For all but two electrode impedance measurements, the electrode impedance values remained under 100 kΩ and for most electrodes they remained under 50 kΩ.

#### 3.2.2. Eye-Tracking Test

**Figure 6:**
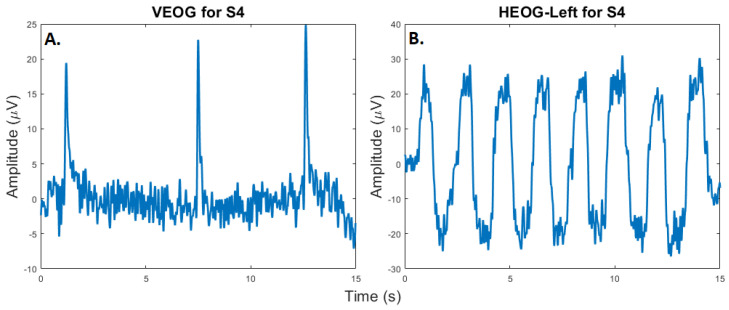
(**A**). **Eye blinks:** A participant (S4) was instructed to blink three times during a session. The plot shows the signal detected by the vertical EOG sensor. (**B**). **Eye Movements:** The same participant was instructed to move her eyes left–to–right and right–to–left over a period of 15 s. The resulting plot shows the oscillating EEG due to these repetitive eye movements.

In this test, we recorded eye blinks and horizontal and vertical eye movements from a center position using the GUI. Examples of eye blinks and tracking of eye movements, which were acquired at 80 Hz, are presented in Figure 6. Measurements of eye movements and eye blinks are critical for the identification and removal of ocular artifacts from EEGs in BCI systems or for use as additional signal sources for control. In our proposed system, H-infinity adaptive noise cancellation, an adaptive filtering technique that requires representations of the EOG signals, is implemented on board for real-time operation [5].

#### 3.2.3. Synchronized EEG–EOG–IMU Test

**Figure 7:**
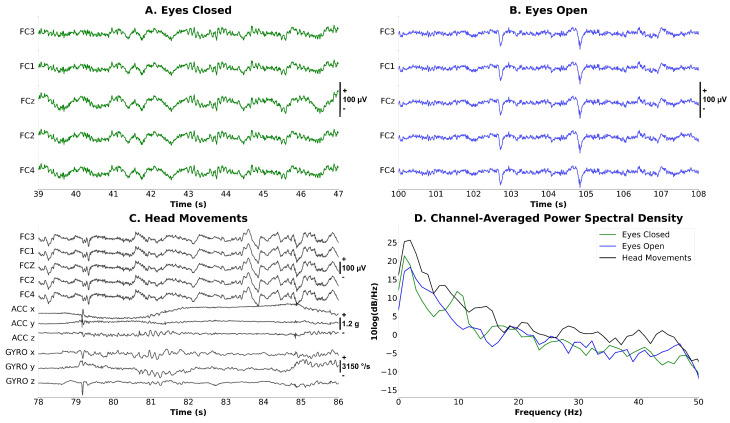
**Characterization of EEG in three task conditions**. (**A**). **Eyes Closed (EC):** A participant was instructed to maintain eyes closed for a period of 8 s during the session. (**B**). **Eyes Open (EO):** The participant maintained eyes open for a period of time. (**C**). **Head Movement (HM):** The participant was asked to move the head towards the front, back, left, and right for a period of time. The resulting plot demonstrates correct synchronization of EEG and IMU data based on the resulting movement artifacts in the EEG signal. (**D**). **Spectral Comparison between EO, EC, and HM conditions.**

The synchronized acquisition of EEG, EOG, and IMU data from the user’s head is important for characterizing head movement and the identification and removal of potential motion artifacts from the EEG signals [6]. Figure 7 depicts raster plots of EEG measurements acquired during conditions with (A) eyes closed, (B) eyes open, and (C) head movements collected at 80 Hz. A band-pass filter from 1 Hz to 50 Hz was applied to the signals and no additional de-noising methods were employed. Figure 7C depicts a raster plot showing synchronized EEG and IMU recordings during head movements towards the front, back, left, and right for one participant. As expected, the head motion, as displayed by the IMU channels (e.g., ACC and GYRO), coincides with motion artifact contamination of the EEG data. Additionally, as compared to the eyes-open and eyes-closed conditions, EEGs during head movement experience an increase in gamma activity due to EMG contamination, which matches the prior literature on EMG contamination during head movement (see Figure 7D) [76].

Figure 7D depicts the spectral characteristics of EEG during eyes-open, eyes-closed, and head-movement conditions. These spectral characteristics demonstrate the 1/f spectrum typical of EEG signals. Moreover, the EEG during the eyes-closed condition shows a modest increase in alpha (8–12 Hz) power as compared to the eyes-open condition [77] as the electrodes are positioned over motor areas rather than occipital areas where large alpha waves would be expected.

#### 3.2.4. Open-Loop Performance

**Figure 8:**
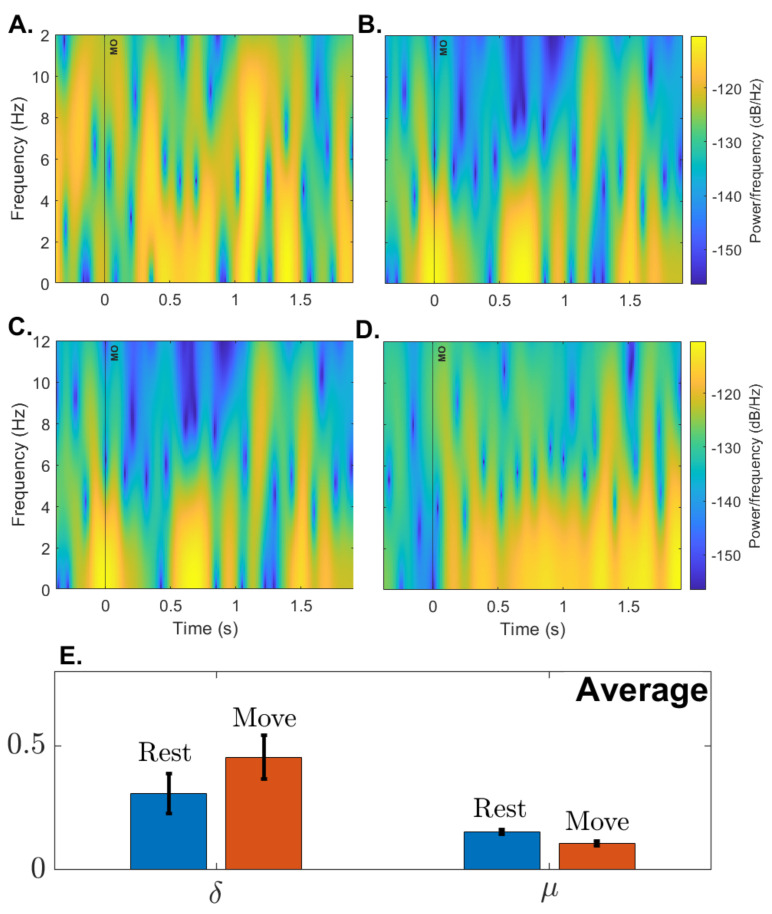
**Spectrogram and relative power:** (**A**–**D**). Plots show the average spectrogram for participants S1–S4 from 0.5 s before movement onset (MO) to 2 s after MO. (**E**). shows average relative power in the δ and μ frequency bands among participants. The average is based on two blocks with twenty trials each.

To further assess the spectral characteristics of EEG, four participants underwent two blocks of 21 trials of a simple GO–NOGO paradigm. In this paradigm, the system’s user interface first asked the participant to fix their attention on a cross (NOGO) for five seconds. The user’s interface then presented a circle and indicated to the user to move their arm from a horizontal to a vertical position (GO). The expected spectral trend for a paradigm of this nature [78,79,80] would be that, when moving from NOGO to GO, the relative power in the δ band should increase while the relative power in the μ band should decrease. Figure 8 shows that the relative power in these two bands for all participants follow our expectations. The paired *t*-tests with Rest/Move factor for all electrodes, except $FC_{4}$, were significant (*p* < 0.0001) : t(167)=11.8, *p* = $9.3\times 10^{-24}$ for δ, and t(167)=−9.3, *p* = $7.0\times 10^{-17}$ for μ.

### 3.3. Closed-Loop BCI Validation

In this section, we report the findings from closed-loop BCI assessments, including IoT functionality and BCI decoder training and performance. The closed-loop BCI validation was designed based on the BCI–robot neurorehabilitation study described in [17,81] and tested on an individual diagnosed with chronic stroke. A significant difference is that testing of the participant was carried out first at the clinic and then at his home, as described below.

#### 3.3.1. IoT Functionality Test

**Figure 9:**
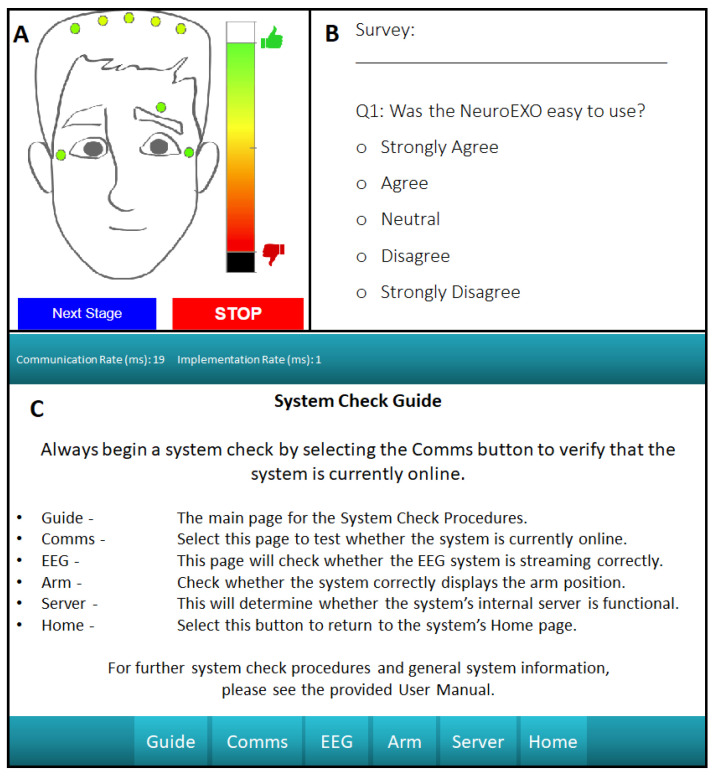
(**A**): User-friendly interface that presents real-time impedance measurements. (**B**): Easy-to-use survey functionality for direct user feedback. (**C**): Debugging interface that can be used for troubleshooting of the system by the user, including a real-time metric for the communication rate between the system and the selected tablet.

A general BCI system must be able to interact with a wide range of IoT-enabled devices. In this example deployment, the system’s communication rate via WiFi was verified in two ways: communication with a robot rehabilitation device and with several different WiFi-enabled tablets for the visual (GUI) display. In this test, communication with the rehabilitation device was found to remain under 50 ms. The displays and browsers tested include the iPhone (7+ or greater) and an Amazon Fire tablet with the Google Chrome, Microsoft Edge, and Amazon Silk browsers, with all tested browsers and displays able to maintain a communication rate under 25 ms. Figure 9 presents the GUI developed for this example deployment and the means to assess the real-time communication rate between the tablet and the system.

#### 3.3.2. Support Vector Machine Model Training

**Figure 10:**
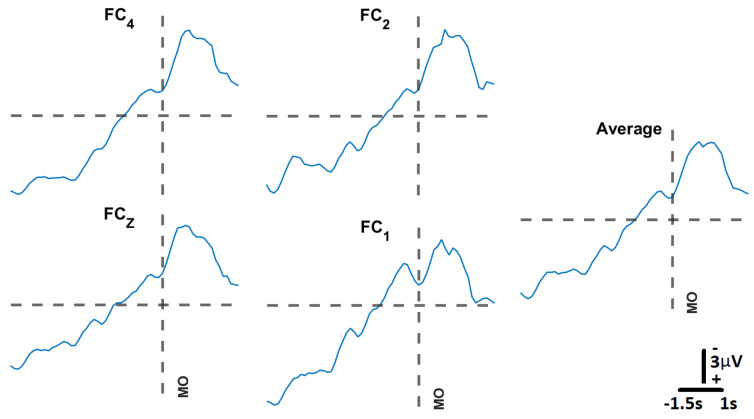
**Movement–related cortical potential, MRCP:** Following the protocol proposed by [17], we obtained the MRCP for participant S005. For each channel, the MRCPs were obtained from averaging 20 trials. The spatial average of those averages is the plot labeled “Average”. Channel FC${}_{3}$ was excluded due to its high impedance value for this participant. The vertical broken line represents the movement onset (MO).

Figure 10 presents the movement-related cortical potentials (MRCPs) recorded through the experimental protocol, which were then used to train the SVM neural decoding model. Table 4 presents the decoding accuracies on S005’s data for a model trained with the hyperparameters displayed in Table 4, where the rejection rate refers to the amount of outliers in the data to be rejected for the training and validation of the model. All models were trained with four-fold cross-validation.

#### 3.3.3. Closed-Loop BCI Performance

**Figure 11:**
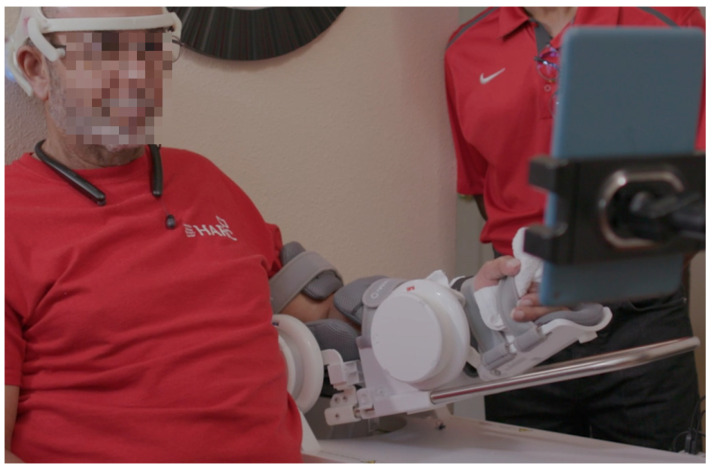
The closed-loop BCI–robot neurorehabilitation system in use at the home of the participant with chronic stroke.

**Figure 12:**
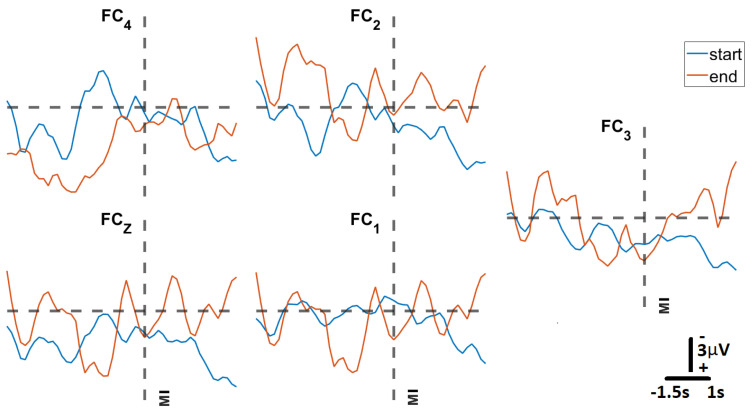
**Average MRCP amplitudes at start and end of therapy:** The subplots present MRCPs across each of the five EEG electrodes recorded for participant S005 at start (block 1) and end (block 105) after six weeks of the at-home BCI therapy. Each MRCP is the result of averaging each of the 20 trials in each block. The vertical dotted line represents the moment movement intent (MI) was detected by the trained SVM machine learning model.

To assess whether the trained SVM could correctly predict motor intent during closed-loop BCI operation, the system was deployed during a series of GO (Move)–NOGO (Fixate) trials at the participant’s home after initial calibration in a clinical setting (Figure 11). For this test, the participant underwent two sessions per day, with each session consisting of three blocks of 20 trials over a period of six weeks and an average of six sessions per week. In Figure 12, we present signals classified by the trained model as representative of motor intent, where “Movement Intent” indicates when the model detected the participant’s motor intent using MRCPs. Each of these signals is the average of the 20 trials from the first block at the start of the protocol (in blue) and the last block at the end of the protocol (in orange). We can see here how the MRCP evolves across the six weeks of at-home BCI therapy for four of the five EEG channels (FC${}_{4}$, FC${}_{2}$, FC${}_{Z}$, FC${}_{1}$). This evolution is not evident in the case of $FC_{3}$, which is the result of the relatively poor contact between that channel and the scalp of the participant, which had impedance values of greater than 100 kΩ).

## 4. Discussion and Conclusions

The design and validation of a custom EEG-based closed-loop BCI headset with onboard processing capabilities has been presented in this report. The design criteria required the consideration of a number of factors. Here, we have developed a minimal viable solution to this design task that is low-cost, portable, wireless, and easy to use and has high interoperability. To ensure a comfortable user experience, the proposed solution has a form factor that provides a one-size-fits-all approach and includes a user-friendly graphical interface for use at home. Additionally, the system has real-time adaptive signal de-noising and decoding capabilities built into the onboard processing board, making the system fully contained within the headset, a feature not currently found in off-the-shelf commercially available systems. All components of the system have been extensively bench tested and also validated with healthy adults, including an individual with chronic stroke.

In the development of the proposed system, the importance of understanding the cascading nature of single design decisions cannot be overstated. Early design decisions can significantly impact the available options for hardware and software functionality and overall system operation. For the current system, the most influential design choice was in the selection of LabVIEW as the back-end coding language. While LabVIEW has a large number of well-tested libraries available, many of these libraries require a processing board developed by National Instruments. Due to the cost of those boards, the selection of the processing board was limited by whether the board was capable of using an open-source user-built LabVIEW library, which is not as well-tested as the libraries developed by National Instruments. Many of the challenges faced in the development of the proposed system were due to incompatibilities between LabVIEW and the low-cost processing board. Careful selection of the high-level system components (such as the backend language, port selections, wireless protocol, etc.) are critical for maximizing the performance and flexibility. In this regard, and to show the flexibility of our proposed system, we have recently programmed the board in C++ and achieved an open-loop sampling rate of 250 Hz.

In conclusion, the proposed system should provide an open test bed for developing low-cost and portable yet effective custom EEG-based closed-loop BCI systems with wireless capabilities, which will help expand the potential user base and application domains and increase the feasibility for academic research and workforce development.

## Figures and Tables

**Table 1:** Engineering specifications for the proposed closed-loop BCI device.

**Headset Specifications**	
Circumference Adjustment Range (cm)	52.3–61.2
Head Breadth Adjustment Range (cm)	13.8–16.6
Head Length Adjustment Range (cm)	17.3–21.4
Electroencephalography (EEG) Electrode Locations	Frontocentral (FC) 3, FC1, FCz, FC2, FC4
EEG Electrode Type	Dry Comb Electrodes
Electrooculography (EOG) Electrode Locations	Both Temples, Above Left Eye
Reference Electrode Locations	Mastoids
EOG and Reference Electrode Type	Dry Flat Electrodes
**Amplifier Specifications**	
Number of Channels	8
Signal–to–Noise Ratio (SNR) (dB)	121
Input Noise ($\mu V_{\mathrm{PP}}$)	1.39
Common–Mode Rejection Ratio (CMRR) (dB)	110
Analog–to–Digital Converter (ADC) Resolution (bits)	24
Impedance (MΩ)	1000
Maximum Sampling Rate (Hz)	500
Bandwidth (Hz)	DC-131
Input range (mV)	±104
Resolution (μV)	0.012
**Inertial Measurement Unit Specifications**	
ADC	16
Gyro Full-Scale Range (dps)	250–2000
Acc Full-Scale Range (g)	2–16
Zero offset error (for 250 dps)	5
Zero-g Offset (mg)	±50
Power Consumption Acc+Mgn (mW)	0.58
Power Consumption Gyro (mW)	4.43
**Brain–Computer Interface Specifications**	
Processor Speed (GHz)	1
Processor Memory (MB)	512
Processor Storage (GB)	4
Open-Loop Sampling Frequency (Hz)	80
Closed-Loop Sampling Frequency (Hz)	40
Communication	802.11 b/g/n WiFi
Backend Coding Language	LabVIEW
Frontend Coding Language	JavaScript (JS), Cascading Style Sheets (CSS), HyperText Markup Language (HTML)
Machine Learning Capability	Support Vector Machine
De-noising Capabilities	Low- and High-Pass Filters; Adaptive Noise Cancellation
Battery Capacity (kWh)	2.96

**Table 2:** Bench testing and human-subject validation methodology.

Headset Design Validation		
**Test Name**	**Description**	**Assessment Tool/Specifications**
System Comfort	Evaluation of user’s comfort level	Questionnaire/Likert scale
System Usability	System Usability Scale (SUS) [28]	SUS > 65 [67]
**Open-Loop BCI Validation**		
Test Name	Description	Target Specifications
Signal Quality	Assessment of electrode and skin sensor impedance	Impedance < 100 kOhm
Eye Tracking	EOG evaluation	Detection of eye blinks and eye movements
Synchronized EEG-EOG-IMU	Acquire multi-modality data streams to confirm synchronized streaming of data	Synchronized EEG-EOG-IMU recordings ≤ 4 ms
Open-loop BCI Performance	Assessment of EEG power modulations in delta and mu bands during a GO-NOGO task	Event-related desynchronization/synchronization (ERD/ERS)
**Closed-Loop Brain–Computer Interface Validation**		
Test Name	Description	Target Specifications
IoT Functionality	Assess communication rates between the headset and multiple types of devices	Communication rate < 50 ms for all connected devices
SVM Model Training	Evaluation of decoding accuracy for motor intent	Model accuracy ≥ 80%; detection of MRCPs
Closed-loop Performance	Evaluation of trained SVM for online prediction of motor intent	≤50 ms closed-loop performance

**Table 3:** Comfort Score: 1: “Strongly Agree” to 5: “Strongly Disagree”.

Participant #	“Moving”	“Dents”	“Too Big”	“Too Small”
S1	5	5	5	5
S2	5	2	5	5
S3	4	2	3	3
S4	4	2	5	5
S5	5	3	5	5
Mean	4.6	2.8	4.6	4.6
SD	0.548	1.304	0.894	0.894

**Table 4:** **Hyperparameter optimization:** closed-loop model hyperparameter optimization using 4-fold cross-validation on participant S005’s data.

Hyperparameter Optimization		
**Rejection Rate**	**Channels Not Used**	**Accuracy**
0	-	85.5%
0.1	-	97.4%
0.323	-	100.0%
0.3	-	100.0%
0	FC3	96.3%
0.1	FC3	98.6%
0.2	FC3	99.3%
0.3	FC3	99.1%

**Figure A1:**
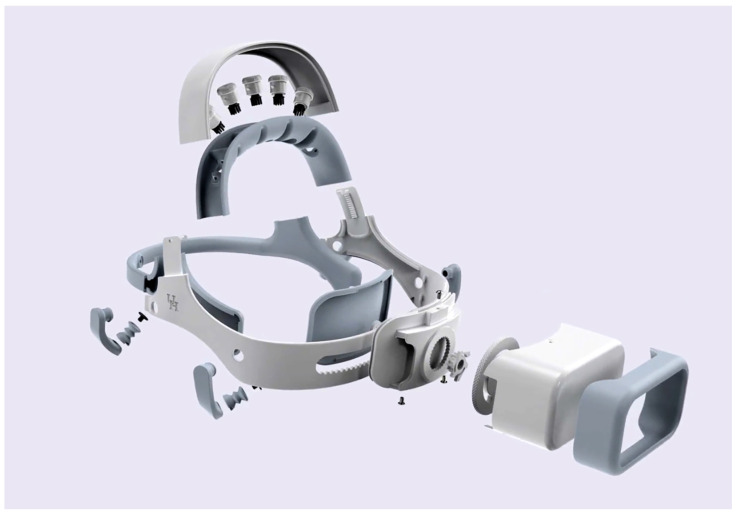
The headset exploded view showing all components. This figure was adapted from US provisional patent #62857263.

## Data Availability

Data is available upon reasonable request by contacting the corresponding author.

## Appendix A

### Appendix A.1. Inertial Measurement Unit Characteristics

**Table A1:** Inertial measurement unit: ICM-20948 [62].

Metric	ICM-20948
ADC (bits)	16
Dynamic Range (dps)	250–2000
Zero offset error (dps) (at 250 dps)	±5
Zero-g Offset (mg)	±50
Power Acc + Mgn (mW)	0.58
Power Gyro (mW)	4.43
